# Hypoxia promotes Rab5 activation, leading to tumor cell migration, invasion and metastasis

**DOI:** 10.18632/oncotarget.8794

**Published:** 2016-04-18

**Authors:** Patricio Silva, Pablo Mendoza, Solange Rivas, Jorge Díaz, Carolina Moraga, Andrew F.G. Quest, Vicente A. Torres

**Affiliations:** ^1^ Institute for Research in Dental Sciences, Faculty of Dentistry, Universidad de Chile, Santiago, Chile; ^2^ Advanced Center for Chronic Diseases (ACCDiS), Faculty of Medicine, Universidad de Chile, Santiago, Chile; ^3^ Center for Molecular Studies of the Cell (CEMC) and Program of Cell and Molecular Biology, Institute of Biomedical Sciences, Faculty of Medicine, Universidad de Chile, Santiago, Chile

**Keywords:** Rab5, hypoxia, migration, metastasis, tumor

## Abstract

Hypoxia, a common condition of the tumor microenvironment, is associated with poor patient prognosis, tumor cell migration, invasion and metastasis. Recent evidence suggests that hypoxia alters endosome dynamics in tumor cells, leading to augmented cell proliferation and migration and this is particularly relevant, because endosomal components have been shown to be deregulated in cancer. The early endosome protein Rab5 is a small GTPase that promotes integrin trafficking, focal adhesion turnover, Rac1 activation, tumor cell migration and invasion. However, the role of Rab5 and downstream events in hypoxia remain unknown. Here, we identify Rab5 as a critical player in hypoxia-driven tumor cell migration, invasion and metastasis. Exposure of A549 human lung carcinoma, ZR-75, MDA-MB-231 and MCF-7 human breast cancer and B16-F10 mouse melanoma cells to hypoxia increased Rab5 activation, followed by its re-localization to the leading edge and association with focal adhesions. Importantly, Rab5 was required for hypoxia-driven cell migration, FAK phosphorylation and Rac1 activation, as shown by shRNA-targeting and transfection assays with Rab5 mutants. Intriguingly, the effect of hypoxia on both Rab5 activity and migration was substantially higher in metastatic B16-F10 cells than in poorly invasive B16-F0 cells. Furthermore, exogenous expression of Rab5 in B16-F0 cells predisposed to hypoxia-induced migration, whereas expression of the inactive mutant Rab5/S34N prevented the migration of B16-F10 cells induced by hypoxia. Finally, using an *in vivo* syngenic C57BL/6 mouse model, Rab5 expression was shown to be required for hypoxia-induced metastasis. In summary, these findings identify Rab5 as a key mediator of hypoxia-induced tumor cell migration, invasion and metastasis.

## INTRODUCTION

Metastasis is a multifactorial process that depends on a variety of cellular traits, including enhanced cell migration and the ability to invade surrounding tissues [1]. Accumulating evidence indicates that tumor cell migration and invasion are strongly influenced by microenvironmental cues, such as the composition of the extracellular matrix, the lack of nutrients and changes in oxygen availability [2, 3]. Particularly, low oxygen tension or hypoxia is associated not only with poor patient prognosis [4, 5], but also with metastasis by stimulating events leading to angiogenesis and tissue invasion [6]. The mechanisms implicated in hypoxia-induced metastasis include those dependent on and independent of the Hypoxia Inducible Factor (HIF) [7, 8], a transcription factor that is stabilized during hypoxia and promotes the expression of a wide array of genes implicated in cell adaptation [7]. Alternatively, hypoxia stimulates tumor cell migration and invasion via increased activation of matrix metalloproteinases, remodeling of the extracellular matrix [3], activation of signaling molecules including Focal Adhesion Kinase (FAK) and Rac1 [9–11], and by activating pathways initiated by growth factors [12], amongst other events (Reviewed in [6]). Interestingly, several of these processes initiated by hypoxia are known to be affected by alterations in endosomal proteins [13]. In particular, hypoxia has been shown to promote re-localization of Rab11-positive recycling endosomes in a microtubule-dependent manner, leading to increased surface expression of integrin α_6_β_4_ and invasion of breast tumor cells [14]. These observations are intriguing, as changes in expression or activity of Rab proteins and their effectors have been identified as a recurrent phenomenon in cancer [15, 16]. However, besides the aforementioned studies, the connection between hypoxia and Rab proteins is poorly understood.

Rabs are small GTPases that regulate different aspects of intracellular traffic, including vesicle formation, endosome fusion, sorting, tethering and transport along microtubules [17]. Given their pleiotropic functions, several Rabs have been shown to be either up-regulated or down-regulated in cancer [18]. In this respect, the early endosome protein Rab5 is particularly interesting, because it is required for different aspects of tumor cell metastasis [19]. Rab5 promotes cell migration and invasion by stimulating focal adhesion (FA) turnover [20, 21], FAK phosphorylation [20], Rac1 activity [22, 23] and matrix metalloproteinase release/activation [20, 24]. Most of these events are dependent of Rab5 functional activity (i.e. GTP-loading), which has encouraged different groups to identify intracellular pathways involved in both Rab5 activation and tumor cell migration and invasion [21–23, 25–27]. However, despite the widely recognized effect of microenvironmental cues on tumor cell dissemination, the role of extra-cellular stimuli on Rab5 function remains unexplored. In particular, the potential connection between hypoxia and Rab5 is an intriguing possibility, as both are known to promote tumor cell migration, invasion and metastasis by similar signaling mechanisms. In this study, we show that hypoxia stimulates Rab5 activity in a HIF-1α-dependent manner, and induces re-localization of Rab5 to FAs in tumor cells. Interestingly, hypoxia was found to increase FAK phosphorylation, Rac1 activity and tumor cell migration in a Rab5-dependent manner. Finally, Rab5 was shown to be required for invasion and metastasis induced by hypoxia.

## RESULTS

### Hypoxia promotes Rab5-GTP loading and re-localization in tumor cells

**Figure 1:**
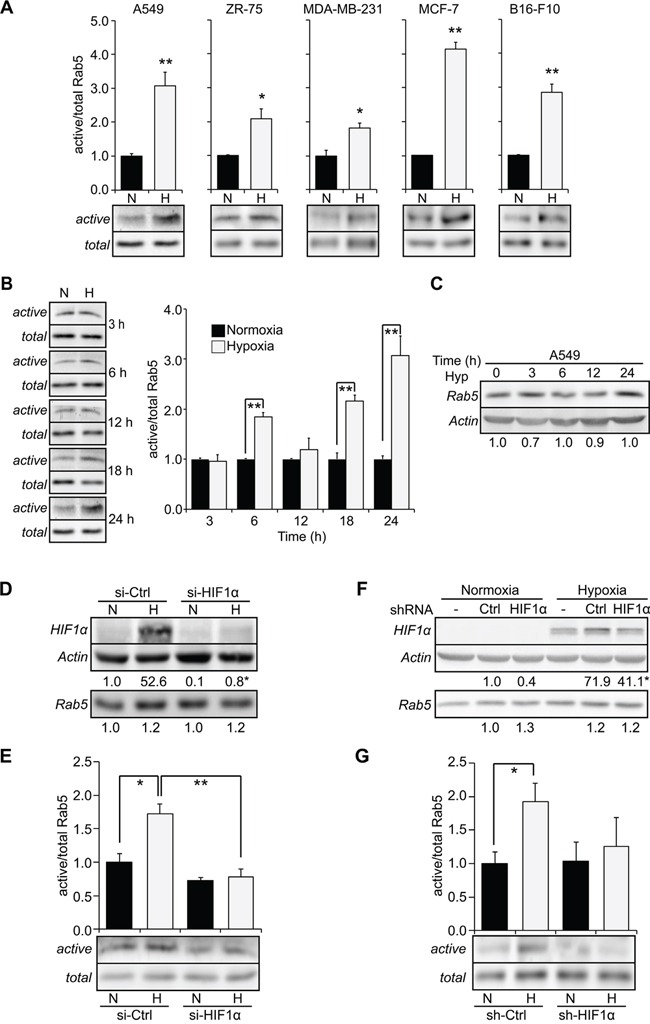
Hypoxia promotes Rab5-GTP loading in tumor cells **A.** A549, ZR-75, MDA-MB-231, MCF-7 and B16-F10 cells were incubated in normoxia (N) or hypoxia (1% O_2_, H) for 24 hours and whole cell lysates were prepared. Rab5-GTP levels were determined by the R5BD pull-down assay. *Lower panels*, representative Western blot images from four independent experiments are shown. *Upper graphs*, relative Rab5-GTP levels normalized to total Rab5 by scanning densitometry are shown as the fold increase with respect to normoxia. Data represent the average of four independent experiments (mean ± s.e.m.). *P<0.05; **P<0.01. **B.** A549 cells were incubated in normoxia (N) or hypoxia (H) for different time periods as indicated, and Rab5-GTP levels were determined, as indicated in (A). *Left panels*, representative Western blot images from four independent experiments are shown. *Right graph*, relative Rab5-GTP levels were determined as indicated in (A). Data represent the average of four independent experiments (mean ± s.e.m.). **P<0.01. **C.** A549 cells were exposed to hypoxia (1% O_2_) for 0, 3, 6, 12 and 24 hours, and then, whole cell lysates were prepared and analyzed by Western blotting. Rab5 and actin were detected by immunoblotting. Representative images are shown. Numerical data below panel represent the scanning densitometry analysis of Rab5 levels normalized to actin from four independent measurements (mean ± s.e.m.) and are summarized as follows: normoxia (1.0 ± 0.04), hypoxia 3 hrs (0.7 ± 0.08), hypoxia 6 hours (1.0 ± 0.08), hypoxia 12 hours (0.9 ± 0.06), hypoxia 24 hours (1.0 ± 0.1). It should be noted that homogenization protocols were different in B and C (for details, see materials and methods), and hence the time course experiments cannot be combined in a single panel. **(D, E)** A549 cells were transfected with control or HIF-1α targeting siRNA constructs and exposed to normoxia or hypoxia for 24 hours. **D.** Whole cell lysates were prepared and analyzed by Western bloting with antibodies against HIF-1α, Rab5 and actin. Representative images are shown. Numerical data below each panel represent the average from three experiments evaluated by scanning densitometry analysis of HIF-1α and Rab5 levels, normalized to actin, and summarized as follows. HIF-1α: si-Ctrl normoxia (1.00 ± 0.01), si-Ctrl hypoxia (52.6 ± 4.7), si-HIF-1α normoxia (0.10 ± 0.05), si-HIF-1α hypoxia (0.75 ± 0.51). Rab5: si-Ctrl normoxia (1.00 ± 0.23), si-Ctrl hypoxia (1.19 ± 0.40), si-HIF-1α normoxia (1.04 ± 0.15), si-HIF-1α hypoxia (1.24 ± 0.07). *P<0.05, compared to si-Ctrl hypoxia. **E.** Rab5-GTP levels were measured by pull-down. Representative Western blot images are shown. The graph indicates the fold increase, obtained by scanning densitometry analysis and normalized to total Rab5. Data were averaged from three independent experiments (mean ± s.e.m.). *P<0.05; **P<0.01. **(F, G).** A549 cells were stably transfected with control or HIF-1α targeting shRNA constructs and exposed to normoxia or hypoxia for 24 hours. **F.** Whole cell lysates were prepared and analyzed by Western blotting with antibodies against HIF-1α, Rab5 and actin. Representative images are shown. Numerical data below each panel represent the average from three experiments evaluated by scanning densitometry analysis of HIF-1α and Rab5 levels, normalized to actin, and summarized as follows. HIF-1α: sh-Ctrl normoxia (1.00 ± 0.54), sh-Ctrl hypoxia (71.9 ± 0.2), sh-HIF-1α normoxia (0.38 ± 0.16), sh-HIF-1α hypoxia (41.1 ± 1.9). Rab5: sh-Ctrl normoxia (1.00 ± 0.16), sh-Ctrl hypoxia (1.20 ± 0.24), sh-HIF-1α normoxia (1.27 ± 0.08), sh-HIF-1α hypoxia (1.17 ± 0.13). *P<0.05, compared to sh-Ctrl hypoxia. **G.** Rab5-GTP levels were measured by pull-down assays. Representative Western blot images are shown. The graph indicates the fold of increase, obtained by scanning densitometry analysis and normalized to total Rab5. Data were averaged from three independent experiments (mean ± s.e.m.). *P<0.05.

Because hypoxia is known to stimulate the metastasis of tumor cells [6], and Rab5 is an important mediator of tumor cell migration and invasion [19, 24], we evaluated the possibility that a connection may exist between hypoxia and Rab5. To this end, different tumor cell lines, including A549 human lung carcinoma, MDA-MB-231, ZR75 and MCF7 human breast cancer and B16-F10 mouse melanoma cells, were subjected to either normoxia (20% oxygen) or hypoxia (1% oxygen) for 24 hours, and Rab5-GTP levels were measured in pull-down assays. Interestingly, hypoxia induced a substantial increase in Rab5-GTP levels in A549 (3-fold increase), MDA-MB-231 (2-fold increase), MCF-7 (4-fold increase), ZR-75 (2-fold increase) and B16-F10 cells (3-fold increase), when compared to normoxia (Figure 1A). A more detailed analysis in A549 cells revealed that Rab5 activation by hypoxia was biphasic, whereby Rab5-GTP levels peaked after 6 and 24 hours of hypoxia, reaching maximum values at 24 hours (Figure 1B). Intriguingly, augmented Rab5-GTP levels induced by hypoxia were not associated with changes in total Rab5 (Figure 1C), but these changes depended on the Hypoxia Inducible Factor, HIF-1α, since targeting of HIF-1α with either siRNA (Figure 1D, 1E) or shRNA approaches (Figure 1F, 1G) prevented Rab5-GTP loading during hypoxia, without any significant changes in total Rab5 levels.

**Figure 2:**
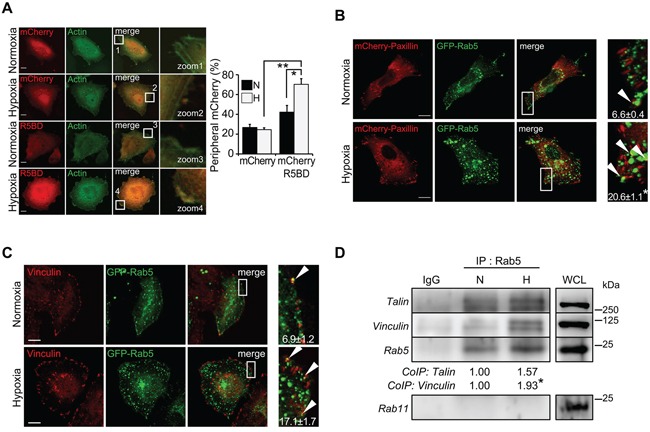
Hypoxia increases the association of Rab5 with focal adhesion proteins **A.** A549 cells were grown on glass coverslips, co-transfected with GFP-actin and the mCherry or mCherry-R5BD constructs previously described [20], and then incubated under normoxic or hypoxic conditions for 24 hours. Samples were fixed and analyzed by confocal microscopy. *Left panels*, representative images are shown. Bar represents 10 μm. *Right panels* are magnifications of boxed areas. *Graph*, the percentage of cells exhibiting peripheral accumulation of mCherry was determined as described in the materials and methods. At least 156 cells per condition were analyzed. Data represent the average of four independent experiments (mean ± s.e.m.). Statistically significant differences are indicated (*P<0.05; **P<0.01). **B.** A549 cells were grown on glass coverslips, co-transfected with mCherry-paxillin and GFP-Rab5, and then incubated in normoxia or hypoxia for 24 hours. Samples were fixed and analyzed by confocal microscopy. Representative images are shown. Bar represents 10 μm. *Right panels* are magnifications of boxed areas. Numbers inside images indicate the Mander's Coefficient, which was obtained from three independent experiments (mean ± s.e.m.). Note that at least 23 images were analyzed per condition. *P<0.05. **C.** A549 cells were grown on glass coverslips, transfected with GFP-Rab5 and then incubated in normoxia or hypoxia for 24 hours. Samples were fixed, incubated with a specific antibody against vinculin (monoclonal antibody) and analyzed by confocal microscopy. Representative images are shown. Bar represents 10 μm. *Right panels* are magnifications of boxed areas. Numbers inside images indicate the Mander's Coefficient, which was obtained from a representative experiment (mean ± s.d.). Note that at least 13 cells were analyzed per condition. **D.** A549 cells were incubated in normoxia or hypoxia for 24 hours and then cell extracts were prepared. Rab5 was immunoprecipitated with a polyclonal antibody and samples were analyzed by Western Blot. For comparison, 50 μg of whole cell lysates (WCL) were analyzed. Control immunoprecipitation experiments were performed with an irrelevant IgG. Relative levels of talin and vinculin were quantified in immunoprecipitates by scanning densitometry of Western Blots and normalized to Rab5 immunoprecipitated and total talin and vinculin (respectively) in WCL. Numerical data below each panel indicates the fold increase in talin (1.57 ± 0.26) and vinculin levels (1.93 ± 0.24) relative to normoxia, as calculated from three independent experiments (mean ± s.e.m.). *P<0.05.

Our previous studies have shown that Rab5 activation in tumor cells is followed by its re-localization to the cell periphery, which is associated with increased cell migration [20, 27, 28]. Thus, we evaluated the localization of active Rab5 during hypoxia, using the mCherry-R5BD construct, which was previously shown to co-localize with Rab5-GTP, but not Rab5-GDP [20]. A549 cells were co-transfected with GFP-actin and mCherry-R5BD or mCherry alone, and then subjected to normoxia or hypoxia for 24 hours. As anticipated, substantial accumulation of mCherry-R5BD, but not mCherry alone, was observed at the periphery of cells exposed to hypoxia (Figure 2A). Of note, re-localization of mCherry-R5BD correlated with peripheral accumulation of endogenous Rab5 (Suppl. Figure 1A), but not other early endosomal proteins (EEA1, early endosome antigen 1, Suppl. Figure 1B) or other Rab proteins, including Rab4 (Suppl. Figure 1C, recycling endosome), Rab21 (Suppl. Figure 1D, early endosome) and Rab7 (Suppl. Figure 1E, late endosome), supporting the selectivity of this phenomenon for Rab5.

### Hypoxia increases the association of Rab5 with focal adhesions and stimulates tumor cell migration

It was previously shown that re-localization of Rab5 to the cell periphery leads to the association with focal adhesion (FA) proteins, including FAK, vinculin and paxillin [20, 27]. Thus, we evaluated the possibility that hypoxia enhances the association of Rab5 with FAs. To this end, confocal microscopy analysis was performed revealing a substantial increase in co-localization between GFP-Rab5 and mCherry-paxillin during hypoxia (Manders coefficient: 6.6 ± 0.4% in normoxia versus 20.6 ± 1.1% in hypoxia, Figure 2B). Similar results were obtained when analyzing the co-localization with vinculin, an endogenous FA marker (Figure 2C). These observations were confirmed by immunoprecipitation experiments, as Rab5 was found to co-immunoprecipitate with vinculin and talin (of note, paxillin antibodies were not suitable for Western Blot analysis) and this association was significantly increased during hypoxia (talin, 1.6-fold increase; vinculin, 1.9-fold increase; Figure 2D). Importantly, other related Rab proteins, including Rab11, failed to co-immunoprecipitate with Rab5 and FA proteins under normoxic and hypoxic conditions (Figure 2D and data not shown).

Hypoxia has been shown to activate FAK (i.e. the phosphorylating activation on Y397, [9]) and tumor cell migration by mechanisms that remain elusive [9, 11]. In agreement with those studies, hypoxia promoted A549 cell migration in wound healing (Suppl. Figure 2A) and Boyden Chamber assays (Suppl. Figure 2B), and stimulated FAK phosphorylation on Y397, as a biochemical readout (Suppl. Figure 2C). Of note, the stimulating effects of hypoxia in both cell migration and Rab5 activity were sustained even after re-oxygenation, suggesting an adaptative response towards hypoxia (Suppl. Figure 2B, 2D).

### Rab5 activation is required for hypoxia-induced cell migration

**Figure 3:**
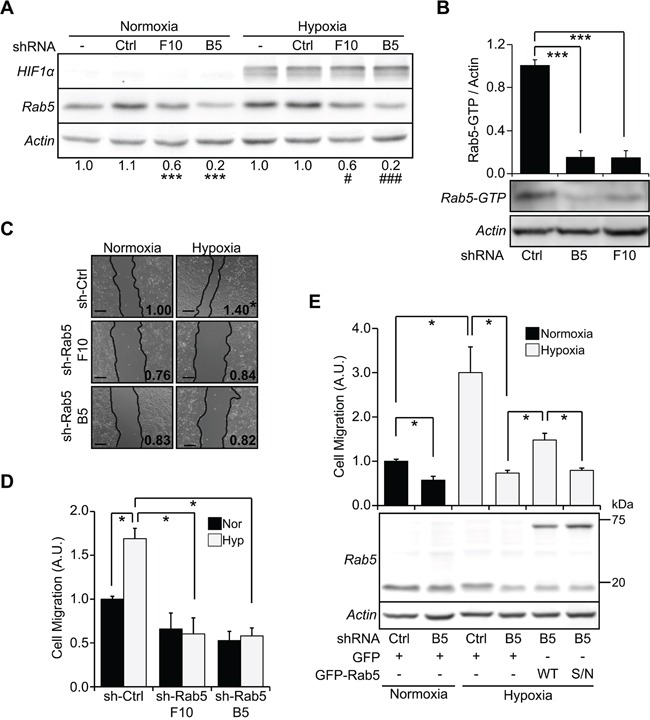
Rab5 activation is required for hypoxia-induced cell migration **A.** A549 cells were stably transduced with either control (sh-Ctrl) or Rab5-specific shRNA constructs (sequences #F10 and #B5) and then, exposed to hypoxia for 24 hours. Whole cell lysates were prepared and analyzed by Western blotting for HIF1α, Rab5 and actin. Numerical data below panel represent the scanning densitometry analysis of Rab5 levels normalized to actin from four independent measurements (mean ± s.e.m.) and are summarized as follows: parental normoxia (1.0 ± 0.05), sh-Ctrl normoxia (1.1 ± 0.03), sh-Rab5#F10 normoxia (0.6 ± 0.03), sh-Rab5#B5 (0.2 ± 0.02), parental hypoxia (1.0 ± 0.1), sh-Ctrl hypoxia (1.0 ± 0.03), sh-Rab5#F10 (0.6 ± 0.08), sh-Rab5#B5 (0.2 ± 0.04). ***P<0.001, compared to sh-Ctrl in normoxia; #P<0.05, ###P<0.01, compared to sh-Ctrl in hypoxia. **B.** A549 cells stably transduced with either control (sh-Ctrl) or Rab5-specific shRNA constructs (sequences #F10 and #B5) were homogenized and Rab5-GTP levels were determined by the R5BD pull-down assay. Representative Western blot images are shown. Graph indicates the fold of increase, obtained by scanning densitometry analysis and normalized to total Rab5. Data were averaged from three independent experiments (mean ± s.e.m.) ***P<0.001. **C.** Cells were grown to confluence, monolayers were wounded and cells were allowed to migrate for 24 hours in normoxia or hypoxia. Representative phase contrast images are shown and numbers within panels indicate the fold increase, which was averaged from four independent experiments (mean ± s.e.m) and is summarized as follows: normoxia/sh-Ctrl (1.00 ± 0.07), hypoxia/sh-Ctrl (1.40 ± 0.1), normoxia/sh-Rab5 F10 (0.76 ± 0.03), hypoxia/sh-Rab5 F10 (0.84 ± 0.05), normoxia/sh-Rab5 B5 (0.83 ± 0.07), hypoxia/sh-Rab5 B5 (0.82 ± 0.05). *P<0.05. Bar represents 200 μm. **D.** Cells were incubated in normoxia or hypoxia for 24 hours, harvested and then allowed to migrate for 2 hours in Transwell chambers coated with 2 μg/ml fibronectin, under normoxic conditions. Cells that migrated were visualized by crystal violet staining. Data represent the average from three independent experiments (mean ± s.e.m.). *P<0.05. **E.** Cells were transiently transfected with shRNA-resistant constructs, pEGFP-C1 (GFP), pEGFP-Rab5 (WT) or pEGFP-Rab5/S34N (S/N), incubated in normoxia or hypoxia for 24 hours, harvested and then allowed to migrate for 2 hours in Transwell chambers coated with 2 μg/ml fibronectin, under normoxic conditions. Transfection efficiency was roughly 40-50%, as determined by flow cytometry (data not shown). Also, whole cell lysates were prepared and analyzed by Western blotting with antibodies against Rab5 (*upper panel*) and actin (*lower panel*). Data represents the quantification of four independent experiments (mean ± s.e.m.). *P<0.05.

Our data indicate that hypoxia promotes Rab5 activation, re-localization to the cell periphery and co-localization with FAs, which is intriguing because the recruitment of Rab5 to FAs was recently shown to precede tumor cell migration and invasion [20]. To evaluate this possibility, endogenous Rab5 was knocked-down by shRNA and cell migration was subsequently determined in wound healing and Boyden Chamber assays. Although the different shRNA sequences used in this study resulted in partial down-regulation of Rab5 (shRNA-B5, 80% decrease; shRNA-F10, 40% decrease), both shRNA sequences provoked a substantial decrease in Rab5-GTP levels to a similar extent (shRNA-B5, 85%; shRNA-F10, 84% decrease) (Figure 3A, 3B). This phenomenon was previously described by our group [20], and is consistent with the notion that a positive-feedback loop for the Rab5 activation cycle exists [29]. Rab5 targeting with both shRNA-B5 and shRNA-F10 inhibited hypoxia-dependent cell migration to a similar extent, as compared to shRNA control cells (Figure 3C, 3D). Importantly, as a control, HIF1α was readily detectable after 24 hours of hypoxia and was not affected by the knock-down of Rab5 (Figure 3A). Our observations showing that Rab5 is activated during hypoxia (Figure 1), along with data indicating that regardless of the effect on total Rab5, both shRNA sequences provoked a similar decrease in Rab5-GTP levels, which paralleled their efficiency in inhibiting cell migration, raised the question as to whether these events are dependent on Rab5 activity. To this end, the response of A549 cells to hypoxia-induced cell migration was evaluated in transfection assays with GFP-Rab5 and the GFP-Rab5/S34N mutant (GDP-bound, inactive Rab5). As expected, transient expression of GFP-Rab5, but not GFP-Rab5/S34N or GFP alone was followed by an increase in cell migration under hypoxic conditions (Figure 3E). Taken together, these data indicate that Rab5 activity is required for hypoxia-dependent cell migration.

### Silencing of Rab5 decreases FAK and Rac1 activation induced by hypoxia, without alterations in endocytosis

**Figure 4:**
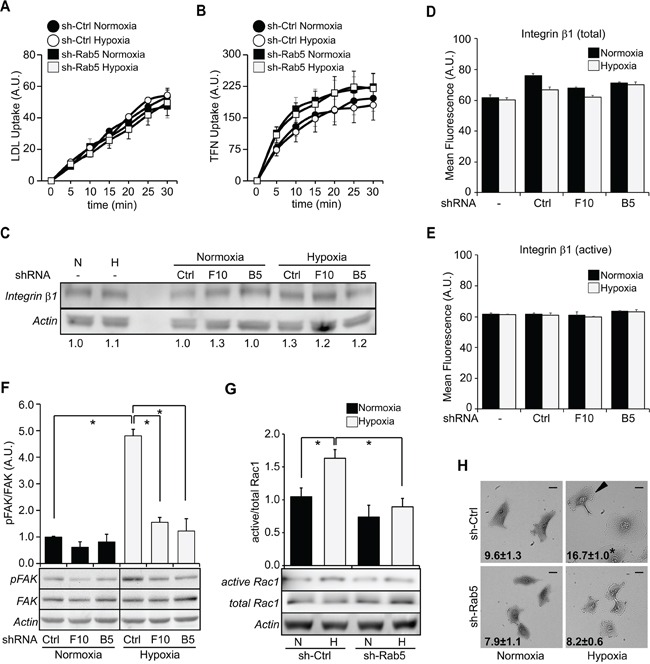
Silencing of Rab5 decreases FAK and Rac1 activation induced by hypoxia, without alterations in endocytosis **A, B.** A549 cells were incubated in normoxia or hypoxia for 24 hours, harvested and incubated with LDL (A) and TFN (B) for internalization assays. Analysis was performed by flow cytometry (for details, see materials and methods). Graphs represent the average of four independent experiments (mean ± s.e.m.). **C.** A549 cells were exposed to normoxia or hypoxia for 24 hours and then, whole cell lysates were prepared and analyzed by Western blotting with antibodies against β_1_ integrin and actin. Representative images are shown. Numerical data below each panel represent the average from two experiments by scanning densitometry analysis of β_1_ integrin levels normalized to actin. **D, E.** A549 cells were incubated in normoxia or hypoxia for 24 hours, harvested, fixed and incubated with antibodies against total (D) or active (E) β_1_ integrin, followed by AlexaFluor488-conjugated secondary antibodies, and surface staining was analyzed by flow cytometry (for details, see materials and methods). Graphs represent the average of values from four independent experiments (mean ± s.e.m.). **F.** A549 cells were exposed to normoxia or hypoxia for 24 hours and then, whole cell lysates were prepared and analyzed by Western blotting with antibodies against FAK, actin and phospho-Y397-FAK (FAK phosphorylated on Y397, pFAK). Representative images are shown. Relative levels of phospho-Y397-FAK were quantified by scanning densitometry and normalized to total FAK. Data represent the average of results from three independent experiments (mean ± s.e.m.). *P<0.05. **G.** A549 cells were exposed to normoxia or hypoxia for 24 hours and Rac1-GTP levels were measured in the GST-PBD pull-down assay. Representative Western blot images are shown. Graph indicates the fold of increase, obtained by scanning densitometry analysis and normalized to total Rac1. Data were averaged from five independent experiments (mean ± s.e.m.) *P<0.05. **H.** Sub-confluent cultures were exposed to normoxia or hypoxia for 24 hours and phase contrast images were recorded by microscopy. Representative images are shown. Bar represents 20 μm. Numbers in panels indicate the percentage of cells depicting a *leading lamellipodium* and a trailing edge (characteristics defined as locomotion structures, arrowhead). Data were obtained from three independent experiments (mean ± s.e.m.). *P<0.05. Note that at least 245 cells were analyzed per experiment.

Because extended exposure to hypoxia was previously suggested to affect endocytosis [13], and given that Rab5 is a central regulator of early endosome dynamics, we evaluated whether hypoxia or Rab5 targeting altered endocytosis of different cargoes. Intriguingly, neither hypoxia nor Rab5 down-regulation affected the internalization of LDL or Tranferrin under these conditions (Figure 4A, 4B), suggesting that the activation of Rab5 during 24 hours of hypoxia does not affect endocytosis in general. Rather, this phenomenon is likely to involve a subset of pathways relevant to cell migration. Since Rab5 is required for β_1_ integrin function [27, 30, 31] and given that most events evaluated in this study depend on this integrin, we evaluated the expression and activation of β_1_ integrin. However, neither total nor surface β_1_ integrin was affected by hypoxia or Rab5 down-regulation (Figure 4C, 4D). Similar results were obtained for surface activated β_1_ integrin (Figure 4E). On the other hand, the signaling molecules Rac1 and FAK represented interesting alternatives, as both are activated during hypoxia [9, 10] and involved in Rab5-dependent cell migration [20, 22, 23]. As anticipated, hypoxia was found to stimulate FAK phosphorylation on Y397, but this increase depended on Rab5, because shRNA-mediated knock-down prevented the increase in phosphorylation of FAK (Figure 4F). It should be mentioned that FAK activity was functional in these cells and was required for hypoxia induced migration A549 cells, because of treatment with the FAK inhibitor PF 562,271 prevented hypoxia-driven cell migration (Suppl. Figure 3A). To further characterize the signaling pathways implicated in Rab5-dependent cell migration induced by hypoxia, we measured the activity of Rac1, which is known to be activated by Rab5 [22, 23]. As predicted, hypoxia induced a substantial increase in Rac1-GTP levels in cells treated with shRNA-control, but not shRNA-Rab5 (Figure 4G). Given that Rac1 is essential for cellular protrusion and the formation of locomotion structures, we further evaluated the formation of leading edge *lamellipodia* during hypoxia and their dependence on Rab5. Indeed, hypoxia enhanced the amount of cells with leading edge *lamellipodia* in shRNA-control, but not shRNA-Rab5 cells (Figure 4H), indicating that Rab5 is necessary for hypoxia-induced Rac1 activation and cell locomotion. Importantly, similar to our previous observations (Figure 3E), expression of GFP-Rab5, but not GFP-Rab5/S34N or GFP alone was sufficient to enhance hypoxia-induced phosphorylation of FAK (Suppl. Figure 3B) and Rac1 activation (Suppl. Figure 3C). Taken together, these data indicate that Rab5 activity is required for FAK and Rac1 signaling induced by hypoxia.

### The aggressiveness of tumor cells determines the magnitude of Rab5 activation and migratory capacity induced by hypoxia

**Figure 5:**
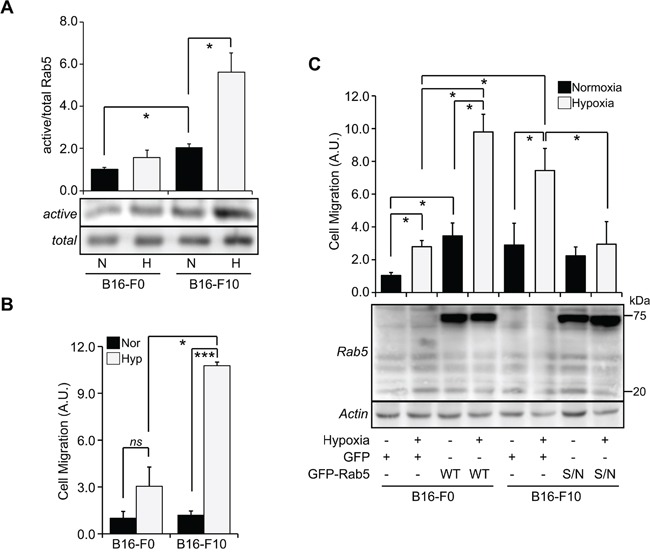
The aggressiveness of tumor cells determines the magnitude of Rab5 activation and migratory capacity induced by hypoxia **A.** B16-F0 and B16-F10 mouse melanoma cells were incubated in normoxia (N) or hypoxia (1% O_2_, H) for 24 hours and whole cell lysates were prepared. Rab5-GTP levels were determined by the R5BD pull-down assay. *Lower panel*, representative Western blot images from four independent experiments are shown. *Upper graph*, relative Rab5-GTP levels were normalized to total Rab5 by scanning densitometry and are shown as the fold increase with respect to normoxia in B16-F0 cells. Data represent the average of four independent experiments (mean ± s.e.m.). *P<0.05. **B.** B16-F0 and B16-F10 cells were incubated in normoxia or hypoxia for 24 hours, harvested and then allowed to migrate for 2 hours in Transwell chambers coated with 2 μg/ml fibronectin, under normoxic conditions. Cells that migrated were visualized by crystal violet staining. Data represent the average from three independent experiments (mean ± s.e.m.). *P<0.05, ***P<0.001. **C.** B16-F0 cells were transfected with pEGFP-C1 (GFP) or pEGFP-Rab5 (WT), and B16-F10 cells were transfected with pEGFP-C1 (GFP) or pEGFP-Rab5/S34N (S/N). Then, cells were incubated in normoxia or hypoxia for 24 hours, harvested and allowed to migrate for 2 hours in Transwell chambers coated with 2 μg/ml fibronectin, under normoxic conditions. Also, whole cell lysates were prepared and analyzed by Western blotting with antibodies against Rab5 (*upper panel*) and actin (*lower panel*). Data represent the quantification of three independent experiments (mean ± s.e.m.). *P<0.05.

Hypoxia is directly associated with the aggressiveness of tumors and it has been shown to be indicative of poor patient prognosis [4]. This raised the question as to whether the effects of hypoxia on Rab5 activity and tumor cell migration depended on the aggressiveness of tumor cells. With this in mind, we evaluated the effect of hypoxia on Rab5 activity and cell migration, by comparing B16-F0 and B16-F10 mouse melanoma cells with low and high metastatic potential, respectively [32]. Intriguingly, hypoxia induced a significant increase in Rab5-GTP levels in B16-F10, but not B16-F0 cells (Figure 5A). Likewise, induction of cell migration by hypoxia was observed in B16-F10, but not B16-F0 cells (Figure 5B). These data suggest that the enhanced sensitivity of metastatic tumor cells towards hypoxia-driven cell migration depends, at least in part, on Rab5 function. To confirm this possibility, we analyzed the effect of expressing different Rab5 mutants in B16-F0 and B16-F10 cells. As suspected, expression of GFP-Rab5 was sufficient to sensitize B16-F0 cells to hypoxia-induced migration, reaching levels similar to B16-F10 cells (Figure 5C). Conversely, expression of GFP-Rab5/S34N prevented the migration of B16-F10 cells exposed to hypoxia (Figure 5C). In agreement with these observations, expression of GFP-Rab5/S34N in B16-F0 failed to increase cell migration induced by hypoxia (data not shown). Taken together, these data suggest that Rab5 expression and its activation are important for the migratory capacity of tumor cells induced by hypoxia.

### Hypoxia promotes tumor cell invasion and metastasis in a Rab5 dependent manner

**Figure 6:**
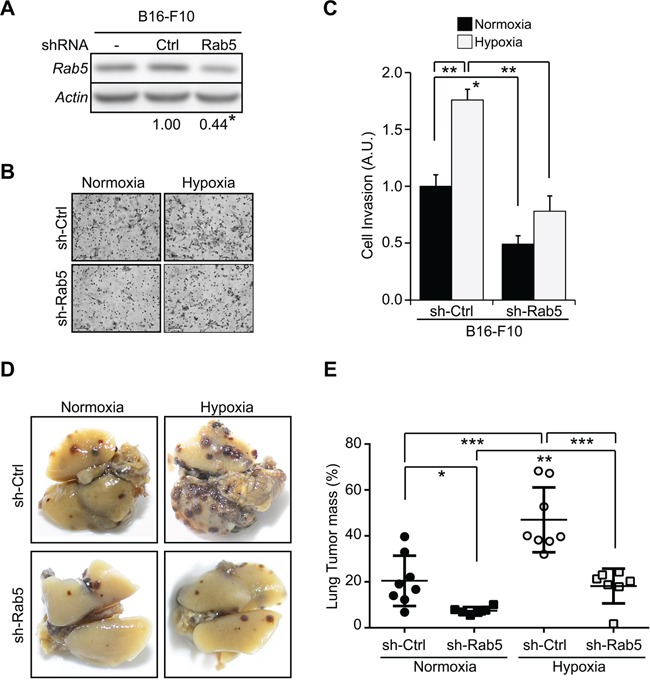
Hypoxia promotes tumor cell invasion and metastasis in a Rab5 dependent manner **A.** B16-F10 cells were stably transduced with either control (sh-Ctrl) or a Rab5-specific shRNA construct (sequence #F8, sh-Rab5). Whole cell lysates were prepared and analyzed by Western blotting. Representative images are shown. Rab5 levels were quantified by scanning densitometry analysis and normalized to actin (numerical data below panel). Residual Rab5 levels in shRNA Rab5 cells (0.44 ± 0.06) were calculated from three independent experiments (mean ± s.e.m.). *P<0.05. **B, C.** B16-F10 cells stably transduced with shRNA constructs were allowed to invade in Matrigel chambers for 24 hours in normoxia or hypoxia. (B) Representative images are shown. (C) Graph represents the quantification of four independent experiments (mean ± s.e.m.). *P<0.05; **P<0.01. **D, E.** B16-F10 cells stably transduced with shRNA constructs were incubated in normoxia or hypoxia for 24 hours. Cells were harvested, re-suspended in physiological saline (2×10^5^ cells) and injected intravenously into the tail vein of C57BL/6 mice. Black lung tumor mass due to metastasis was monitored after sacrificing the animals at 21 days. Representative images are shown in (D). (E) Results are shown for at least 7 mice per group: sh-Ctrl/normoxia, black circles; sh-Rab5/normoxia, black squares; sh-Ctrl/hypoxia, white circles; sh-Rab5/hypoxia, white squares. Statistically significant differences are indicated (*P<0.05; **P<0.01; ***P<0.001).

Since hypoxia promotes not only tumor cell migration, but also invasiveness and metastasis [3, 6, 14, 33], we evaluated the requirement of Rab5 in hypoxia-induced tumor cell invasion and metastasis. To this end, B16-F10 mouse melanoma cells were used, as these cells are suitable for evaluating both cell invasion *in vitro* and metastasis *in vivo*, in the latter case using syngenic C57BL/6 mice as recipients [32, 33]. The requirement of Rab5 for B16-F10 cell invasion and metastasis was evaluated by shRNA-mediated targeting. In doing so, endogenous Rab5 was down-regulated by 56% in B16-F10 cells with a Rab5-specific shRNA construct, as compared with control shRNA (Figure 6A). Using these cells, we found that hypoxia increased matrigel invasion in shRNA-control, but not shRNA-Rab5 cells (Figure 6B, 6C). Finally, the requirement of Rab5 in hypoxia-induced metastasis *in vivo* was evaluated by injecting B16-F10 cells into the tail vein of C56BL/6 mice. To this end, B16-F10 cells transduced with shRNA-control or shRNA-Rab5 were exposed to either normoxia or hypoxia for 24 hours, and then injected into mice. In agreement with previous studies [33], 24 hour-exposure to hypoxia was sufficient to enhance lung metastasis of shRNA-control cells (Figure 6D, 6E). Importantly, Rab5 down-regulation prevented both basal metastasis in normoxic conditions and hypoxia-enhanced metastasis (Figure 6D, 6E). It should be noted that under these conditions, cell proliferation and viability were not affected by either Rab5 knock-down or hypoxia (Suppl. Figure 4). Taken together, these data indicate that Rab5 is necessary for B16-F10 tumor cell invasion and metastasis induced by hypoxia.

## DISCUSSION

We describe here a novel mechanism by which hypoxia promotes tumor cell migration, invasion and metastasis, via activation of Rab5-dependent signaling. Hypoxia promoted Rab5 activation (i.e. GTP loading) in different tumor cells, including A549 lung carcinoma, ZR-75, MDA-MB-231 and MCF-7 breast cancer and B16-10 mouse melanoma cells. Increased Rab5 activation by hypoxia was followed by its re-localization to the leading edge and co-localization with focal adhesions. In fact, Rab5 was found to be required for hypoxia-induced cell migration, FAK activation, Rac1-GTP loading, invasion and metastasis, as shown by shRNA and reconstitution approaches. An important conclusion from our experiments is that the effect of hypoxia on Rab5 varies according to the aggressiveness of tumor cells. Specifically, hypoxia induced a more robust activation of Rab5 in the more aggressive cell line MCF-7, when compared to ZR-75 cells [34]. However, these cells lines are not necessarily comparable, as they are derived from different patients and have been cultured in different conditions [35, 36]. However, in mouse melanoma cells of the same origin [32], hypoxia activated Rab5 in highly metastatic B16-F10, but not in the poorly metastatic B16-F0 cells. These intriguing observations are in agreement with cell migration data, suggesting that the magnitude of Rab5 activation by hypoxia correlates with the migratory capacity of tumor cells. These provocative findings were further confirmed by data showing that the transfection of B16-F0 cells with wild type Rab5, but not an inactive mutant (Rab5/S34N) enhanced their migratory response towards hypoxia, whereas expression of Rab5/S34N in B16-F10 cells prevented hypoxia-induced migration. These *in vitro* observations indicate that the activation of Rab5 by hypoxia increases the migratory capacity of tumor cells and provide the initial proof-of-concept that increasing Rab5 activation in poorly metastatic cells favors the acquisition of traits associated with enhanced metastatic potential. Future studies will be required to substantiate this intriguing possibility.

The effects of hypoxia on Rab5 appeared to be selective, because hypoxia failed to affect the localization of other related Rabs, including early endocytic Rab4 and Rab21 and late endocytic Rab7. Intriguingly, previous studies have shown that hypoxia causes re-localization of Rab11 (which resides in recycling endosomes) to the cell periphery [14]. In agreement with those studies, we observed a partial, but noticeable re-localization of Rab11 in A549 cells exposed to hypoxia (Suppl. Figure 1F). However, no Rab11 or other related Rabs were found to co-immunoprecipitate with Rab5 and FA proteins (Figure 2D, data not shown), further indicating the selectivity of this phenomenon. Moreover, we observed that Rab5 activation after 24 hours of hypoxia is neither related to changes in overall endocytosis of LDL (late endosome cargo) nor transferrin (cargo destined to the recycling pathway). These data are in agreement with previous studies showing that hypoxia induces the endocytosis of cargo proteins after 48 hours, but not after 24 hours [13].

Hypoxia promoted not only the activation of Rab5, but also induced its re-localization and association with the FA proteins paxillin and vinculin, although it remains unclear whether this association occurs at the cell surface or rather within early endosomes that contain FA components, as recently described [37]. This is particularly intriguing, since hypoxia provoked a small, but significant increase in early endosome size, as judged by both Rab5 and EEA1 staining (Suppl. Figure 5). Although we cannot exclude the possibility that Rab5 co-localization with FA proteins occurs within early endosomes, it is worth mentioning that no paxillin or very low levels of vinculin were previously detected in early endosome fractions [37]. Our studies provide evidence that these FA proteins co-localize and co-immunoprecipitate with Rab5 in normoxia and that this association was enhanced during hypoxia. In this respect, previous studies from our group have shown that the association of Rab5 with FAs is followed by FA disassembly [20], leading to the possibility that hypoxia-induced activation of Rab5 accelerates the turnover of FAs, in order to sustain tumor cell migration. Although this possibility remains to be evaluated, this study shows that functionally active Rab5 is necessary for hypoxia-induced migration, FAK phosphorylation on Y397 and Rac1-GTP loading.

Finally, Rab5 was required for tumor cell invasion *in vitro* and metastasis, as shown by matrigel invasion and *in vivo* assays. Using a syngenic mouse model [32], Rab5 down-regulation in B16-F10 cells prevented the metastasis-promoting effect of hypoxia, which was recently documented [33]. It is important to mention that both enhanced migration *in vitro* and metastasis *in vivo* persisted over time and were not affected by re-oxygenation ([33], and this paper). Accordingly, Rab5-GTP levels remained higher after re-oxygenation, suggesting that the activation of Rab5 and the effects on tumor cell migration and metastasis represent adaptive events that may involve *de novo* protein synthesis. In this respect, hypoxia failed to induce changes in total Rab5 levels at the time-points evaluated, although Rab5-GTP loading by hypoxia depended on HIF-1α, suggesting that adaptative mechanisms for Rab5 regulation are induced by hypoxia. An exciting possibility that remains to be evaluated is that proteins controlling either Rab5 activation or inactivation could be regulated by hypoxia, which might explain the maintenance of Rab5 activation during extended periods of hypoxia and following re-oxygenation.

In summary, this work provides evidence for a novel mechanism by which hypoxia promotes tumor cell migration, invasion and metastasis, via activation of Rab5-dependent signaling. The relevance of Rab5 function in tumor cells with different metastatic potential is suggested, as these events are more pronounced in highly metastatic tumor cells. Future studies will be required to substantiate this intriguing possibility.

## MATERIALS AND METHODS

### Materials

Monoclonal anti-Rab5 (sc46692), polyclonal anti-Rab5 (sc28570) and antibodies for Rab4 (sc28569), Rab7 (sc10767), Rab11 (sc9020), Rab21 (sc81917), EEA1 (sc33585), FAK (sc1688) and β_1_ integrin (sc8978) were from Santa Cruz Biotechnology (Santa Cruz, CA). Other antibodies included anti-phospho-Y397-FAK (#3283, Cell Signaling Technology), anti-vinculin (#V4505, Sigma-Aldrich, St. Louis, MO), anti-HIF1α and anti-Rac1 (Transduction Laboratories, Lexington, KY), anti-talin (Chemicon International, MAB1676), and anti-β_1_ integrin (active conformation, Chemicon International, MAB2079Z). Goat anti-rabbit and goat anti-mouse antibodies coupled to horseradish-peroxidase and anti-actin antibody (#A5316) were from Bio-Rad Laboratories (Hercules, CA). Phalloidin-rhodamine, transfection reagent Lipofectamine 2000, AlexaFluor-488 and AlexaFluor-568 secondary antibodies were from Invitrogen (Carlsbad, CA). Tissue culture medium, antibiotics and fetal bovine serum were from GIBCO Life Technologies (Grand Island, NY) and HyClone Laboratories (Logan, UT). Glutathione-Sepharose 4B was from GE Healthcare (Piscataway, NJ). The EZ-ECL chemiluminescent substrate and protein A/G beads were from Pierce Chemical (Rockford, IL). Rab5 lentiviral short hairpin RNAs (shRNA) were from Open Biosystems (Huntsville, AL). The FAK Inhibitor Compound PF562,271 was from Laviana Corporation, as previously described [38].

### Plasmids

The pEGFP-C1 plasmids encoding wild-type Rab5 and Rab5/S34N were kindly provided by Dr. Francisca Bronfman (Pontificia Universidad Católica de Chile, Chile) and previously described [20, 22]. The mCherry-paxillin and pEGFP-C1-actin constructs were kindly provided by Dr. David Schlaepfer (University of California San Diego, USA). The modified pEGFP-C1-mCherry plasmid harboring a deletion of GFP gene sequence, but encoding either mCherry or mCherry-R5BD (the *Rab*5 B*inding* D*omain*, R5BD) were previously described [20].

### Cell culture

A549, MDA-MB-231, MCF-7, ZR-75, B16-F0 and B16-F10 cells were cultured in DMEM-high glucose, DMEM-F12 and RPMI media supplemented with 10% FBS and antibiotics, according to the ATCC's instructions. It should be noted that for most studies, A549 cells were used, because these cells were suitable for down-regulation experiments with different shRNA sequences and for transfection experiments with shRNA-resistant Rab5 mutants. Rab5 down-regulation with different shRNA sequences was previously described [20, 39]. Control cells were infected with a lentivirus encoding a nonspecific shRNA sequence (plasmid 1864; Addgene, Cambridge, MA). Rab5 down-regulation was performed by using the shRNA sequences #B5 and #F10 (Open Biosystems) for A549 cells, and sequence #F8 (Open Biosystems) for B16-F10 cells. HIF-1α was down-regulated both stably with shRNA (Cat N° sc-35561-sh, control shRNA Cat N° sc-108060, Santa Cruz Biotechnology, CA) and transiently with siRNA (Cat N° sc-35561, control siRNA Cat N° 37007, Santa Cruz Biotechnology, CA). Stable cell lines were selected and maintained in 2μg/ml puromycin-containing culture medium. *Transient Transfections*. Cells were grown for 24 hours in complete medium at 50–70% confluence. Transfections were performed with 2 μg of the indicated plasmids (60 mm plates) by using the Lipofectamine 2000 system according to the manufacturer's instructions. Post-transfection, samples were used for analysis by Western blot, fluorescence microscopy and migration assays.

### Hypoxia

Cells were cultured in a hermetically sealed, modular incubator chamber (MIC-101 Billups-Rothenberg Inc., Del Mar, CA). This chamber is a widely used model for inducing hypoxic conditions *in vitro*. The chamber was flushed with a certified gas mixture (1% O_2_, 5% CO_2_ and 94% N_2_, Linde Group®) for 10 minutes at 10 L/min, in order to establish hypoxic conditions, according to the manufacturer instructions. Humidity was reassured in the chamber by placing a plastic petri dish containing 12 mL of sterile water in the chamber. All cells exposed to hypoxia were incubated with hypoxic medium pretreated at least 12 hours prior to assays.

### Western blotting

Cells were washed twice with cold PBS and lysed in 0.2 mM HEPES (pH 7.4) buffer containing 0.1% SDS, phosphatase inhibitors (1 mM Na_3_VO_4_), as well as a protease inhibitor cocktail. Total protein extracts (50 μg/lane, unless indicated) were separated by SDS-polyacrylamide gel electrophoresis and transferred to nitrocellulose membrane. Blots were blocked with 5% milk in 0.1% Tween-PBS and then probed with antibodies. Bound primary antibodies were detected with HRP-conjugated secondary antibodies and the EZ-ECL system.

### Immunofluorescence

Immunofluoresecence was performed as previously described [20]. Vinculin was detected with a monoclonal antibody. Samples were analyzed with a confocal microscope (Carl Zeiss LSM-Pascal 5).

### Rab5-GTP and Rac1-GTP pull down assay

Rab5-GTP and Rac1-GTPpull-down assays were performed as previously described [22, 28]. Briefly, cells were lysed in a buffer containing 25 mM HEPES (pH 7.4), 100 mM NaCl, 5 mM MgCl_2_, 1% NP 40, 10% glycerol, 1 mM dithiothreitol and protease inhibitors. Extracts were incubated for 5 min on ice and clarified by centrifugation (10,000xg, 1 min, 4°C). Post-nuclear supernatants were used for pull-down assays with 30 μg of GSH beads pre-coated with GST-R5BD (Rab5-GTP pull-down) or GST-PBD (Rac1-GTP pull-down) per condition. Beads were incubated with supernatant for 15 min at 4°C in a rotating shaker. Thereafter, beads were collected, washed with lysis buffer containing 0.01% NP 40 and samples were analyzed by Western blotting.

### Immunoprecipitation

Rab5 immunoprecipitation was previously described [20]. This method was developed to minimize GTP hydrolysis, optimizing the recovery of active Rab5, by using short incubation periods. Essentially, cell extracts were prepared in a buffer containing 20 mM Tris, pH 7.4, 150 mM NaCl, 1% NP-40 and protease inhibitors. After incubation for 5 min on ice, samples were centrifuged at 13,000xg for 1 min at 4°C and post nuclear supernatants (500 μg total protein) were immunoprecipitated with protein A/G bead immobilized polyclonal antibodies for no longer than 30 min. Immunoprecipitated samples were solubilized in Laemmli buffer, boiled, separated by SDS-PAGE and analyzed by Western blotting.

### Migration and invasion assays

Cell migration was evaluated in wound healing and Boyden Chamber assays (Transwell Costar, 6.5 mm diameter, 8 μm pore size), whereas invasion was evaluated in Matrigel (BD Biosciences catalog #354480), as previously reported [20, 22]. Cells exposed to hypoxia were harvested and then allowed to migrate in normoxic or hypoxic conditions. Of note, cells were collected in hypoxic pretreated media when migration was evaluated in transwell assays under hypoxic conditions. For wound healing assays, perpendicular cross-shaped wounds were introduced with a p200 pipette tip to monolayers of cells in 24 well plates. All wounded areas were recorded at 40X magnification, by capturing three adjacent images, starting from a distance equivalent to 0.8-1 mm from the cross center (i.e., a total of 12 images were captured per well). Then, plates were placed back into the hypoxic chamber and incubated for 24 h. Wounded areas were recorded at this time point, as described above (12 images per well) and analyzed with *Image J*.

### Cell proliferation and viability assays

Measurements were performed as previously described [39], but important modifications were included. First, experiments were performed in the presence of serum, and second, measurements were done for up to 24 h. *MTS assay*. Cells were seeded on 96-well plates at a density of 1×10^4^ cells per well and incubated for 24 h under normoxic or hypoxic conditions in complete medium. Optical density (O.D.) was assessed with the MTS® kit, by measuring the absorbance at 490 nm, according to the manufacturer's instructions (Promega, Madison, WI, US). *Trypan Blue Exclusion Assay*. Cells were seeded on 24-well plates at a density of 1×10^5^ cells per well and incubated for 24 h under normoxic or hypoxic conditions in complete medium. Then, cells were harvested, resuspended and stained with Trypan Blue for counting in the microscope.

### Image analysis

#### Measurement of the percentage of cells with peripheral mCherry

Measurements were performed as previously described [20]. Cells were transiently transfected with mCherry or mCherry-R5BD and GFP-actin and then exposed to normoxia or hypoxia for 24 hours. After fixation, cells were analyzed by confocal microscopy (Carl Zeiss LSM-Pascal 5). Images were randomly chosen, and entire cells were scored as “positive for peripheral mCherry staining” or “negative for peripheral mCherry staining” based on the detection of mCherry signal at the cell periphery. The percentage of “positive cells” was calculated with respect to the total cell number.

#### Co-localization analysis

Cells were transiently transfected with GFP-Rab5 and mCherry-paxillin and then subjected to normoxia or hypoxia for 24 hours. Co-localization data were evaluated in original images, obtained by Confocal Microscopy (*Carl Zeiss LSM-Pascal 5*). Analysis was performed with the *Image J* software, by using the “*JACoP*” plugin. To this end, the threshold was adjusted in both channels, green for GFP-Rab5 and red for mCherry-paxillin. Additionally, cells were transiently transfected with GFP-Rab5, and vinculin was detected with a specific antibody. In both cases, Mander's coefficients were calculated with respect to GFP-Rab5. At least, 10 images were evaluated per experiment.

### Internalization assays

Cells were grown to 60-80% confluence in complete medium for 24 hours. Thereafter, cells were detached with trypsin, centrifuged, re-suspended in 0.2% BSA in DMEM-high glucose plus 10 mM HEPES, pH 7.4 (internalization medium), and incubated for 30 min at 4°C. For internalization experiments, 250 μl of aliquots of cell suspensions (5×10^5^ cells) were placed in 1 ml tubes. Cells were centrifuged and resuspended with 0.2% BSA in internalization medium containing either 30 μg/μl Alexa Fluor®488-labeled transferrin or 10 μg/μl BODIPY®-FL-LDL at 4°C, for 30 min. Incubations were carried out at 37°C and stopped on ice at different time points as indicated. Then, cells were re-suspended in stripping buffer (0.2% BSA in DMEM-high glucose, pH 3.5, adjusted with HCl). Thereafter, cells were washed with ice-cold PBS, fixed with 0.5% paraformaldehyde, and re-suspended with PBS. Samples were analyzed using a fluorescence activated cell sorter (FACSCanto) and data were analyzed with the WinMDI software.

### Surface β_1_ integrin analysis

Cells were grown for 24 h at sub-confluence in complete medium, under normoxic or hypoxic conditions. Thereafter, cells were resuspended in PBS, fixed with 0.4% paraformaldehyde and blocked in 0.5% BSA/PBS for 30 min at 4°C. Samples were then incubated with the antibodies against active or total β_1_ integrin for 60 min at 4°C, followed by a 30 min incubation with secondary antibodies conjugated to AlexaFluor®488. Finally, cells were resuspended in PBS and analyzed by flow cytometry (FACSCanto; BD Biosciences, Mountain View, CA).

### Syngenic metastasis assay

B16-F10 cells were subjected to normoxia or hypoxia for 24 hours and then injected intravenously into the tail vein of C57BL/6 mice (2×10^5^ cells in 500ul physiological saline solution). After 21 days, mice were sacrificed and lungs were fixed in Feketes solution (70% ethanol, 10% formalin, 5% acetic acid glacial). Black tumor masses were separated from the rest of the lung and weighed. Metastasis was expressed as black tissue mass/total lung mass (%) [40]. The experimental protocols employed were approved by the institutional bioethics committee (CBA 0748 CMUCH).

### Statistical analysis

Where pertinent, results were compared using unpaired t tests with the *GraphPad Prism 5* software (San Diego, CA). Values averaged from at least three independent experiments were compared. A p value < 0.05 was considered significant.

## SUPPLEMENTARY FIGURES


