# Supplementary material for: Hypoxia promotes Rab5 activation, leading to tumor cell migration, invasion and metastasis
Source: Oncotarget. 2016 Apr 18;7(20):29548–62. doi: 10.18632/oncotarget.8794 (PMC5045416; doi:10.18632/oncotarget.8794)
Supplement: Supplementary file 1 [file oncotarget-07-29548-s001.pdf]

SUPPLEMENTARY FIGURES

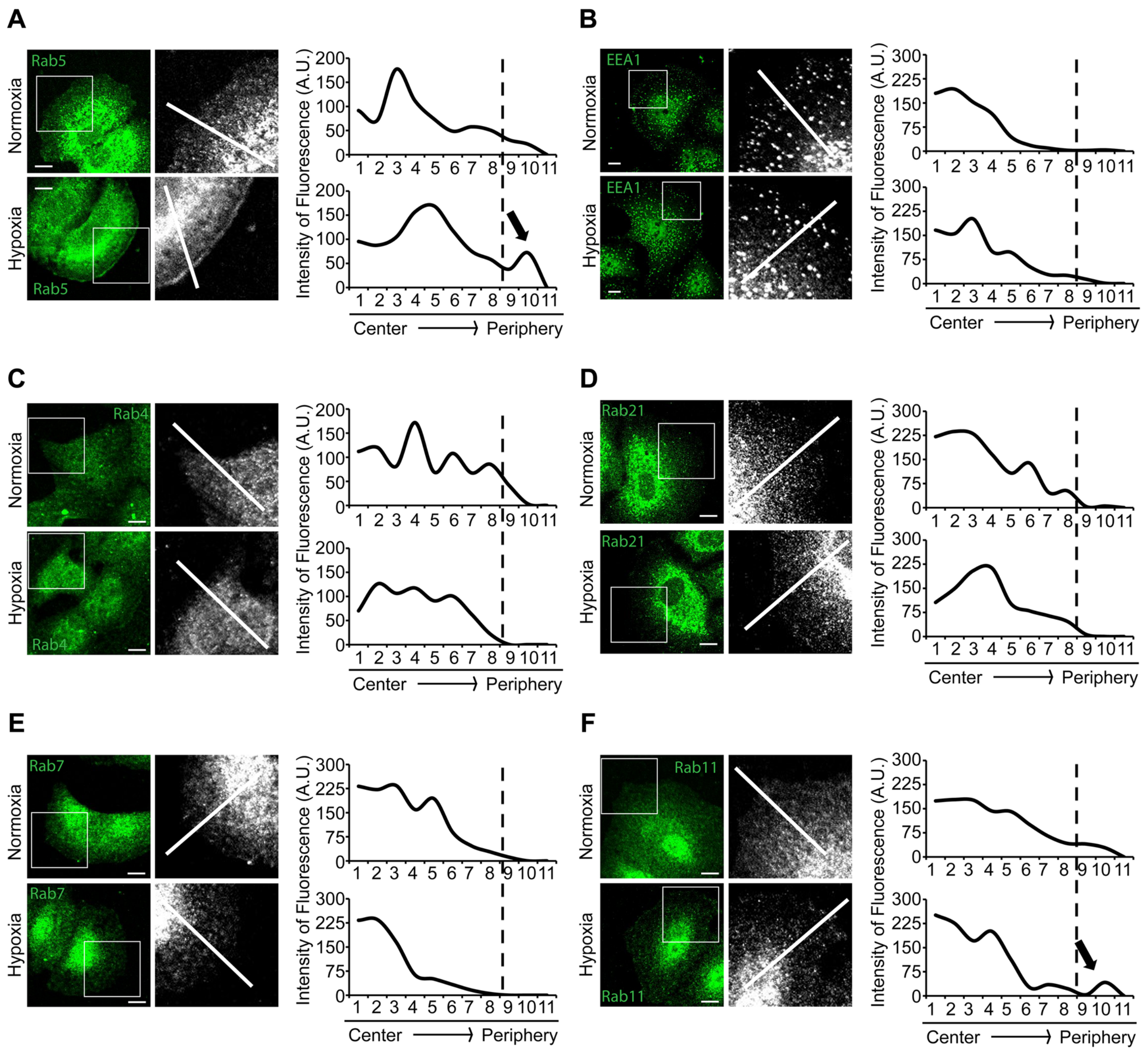

Supplementary Figure S1: A549 cells were grown on glass coverslips and incubated in normoxia or hypoxia for 24 hours. Samples were fixed and incubated with specific antibodies for **A.** Rab5, **B.** EEA1, **C.** Rab4, **D.** Rab21, **E.** Rab7 and **F.** Rab11, and analyzed by confocal immunofluorescence microscopy. Representative images (*left panels*) and magnifications of boxed areas (*middle panels*) are shown. Bar represents 10 µm. *Right graphs* represent the intensity profile plot corresponding to the signal of immunofluorescence for each protein in a set of pixels distributed along the line drawn across the cell (showed in zoomed images). Data are expressed as relative units of fluorescence versus the distance (divided into 10 equal parts, #11 corresponds to background signal). Images are representative of two independent experiments. The cellular periphery (regions 9 + 10) was marked with a dashed line on each graph.

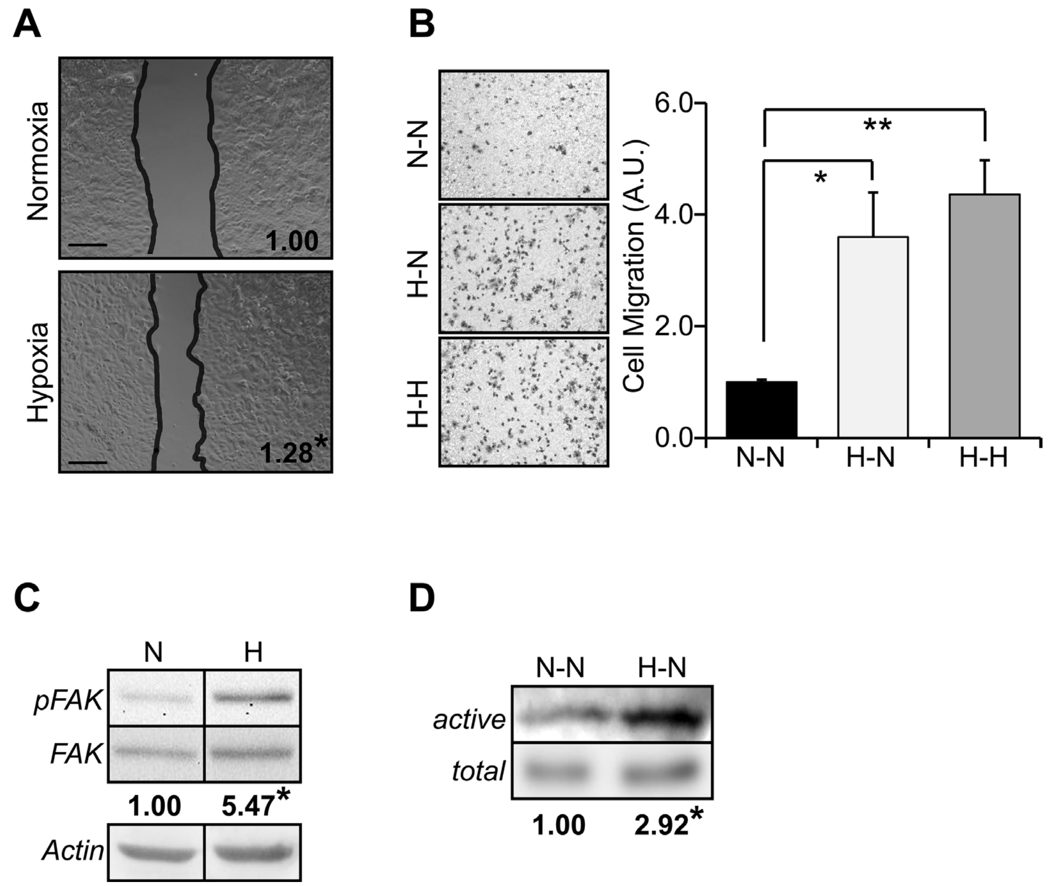

Supplementary Figure S2: A. A549 cells were grown to confluence, monolayers were wounded and cells were allowed to migrate for 24 hours in normoxia or hypoxia. Representative phase contrast images are shown and numbers within panels indicate the fold increase, which were averaged from three independent experiments (mean ± s.e.m) and are summarized as follows: normoxia (1.00 ± 0.06), hypoxia (1.28 ± 0.06) *P<0.05. Bar represents 200 µm. **B.** A549 cells were incubated in normoxia or hypoxia for 24 hours, harvested and then allowed to migrate for 2 hours in Transwell chambers coated with 2 µg/ml fibronectin. Conditions are summarized as follows: 24 hours of normoxia plus 2 hours in transwell under normoxia (N-N); 24 hours of hypoxia plus 2 hours in transwell under normoxia (H-N); 24 hours of hypoxia plus 2 hours in transwell under hypoxia (H-H). Cells that migrated were visualized by crystal violet staining. Data represent the average from three independent experiments (mean ± s.e.m.). *P<0.05; **p<0.01. **C.** A549 cells were exposed to normoxia or hypoxia for 24 hours and then, whole cell lysates were prepared and analyzed by Western blotting with antibodies for FAK, actin and phospho-Y397-FAK (FAK phosphorylated on Y397, pFAK). Representative images are shown. Relative levels of phospho-Y397-FAK were quantified by scanning densitometry and normalized to total FAK. Data represent the average of three independent experiments (mean ± s.e.m.). *P<0.05. **D.** A549 cells were incubated in normoxia for 26 hours (N-N) or 24 hours in hypoxia plus 2 hours in normoxia (H-N), and whole cell lysates were prepared. Rab5-GTP levels were determined by the R5BD pull-down assay. Images representatives of Western blots from three independent experiments are shown. (mean ± s.e.m.). *P<0.05

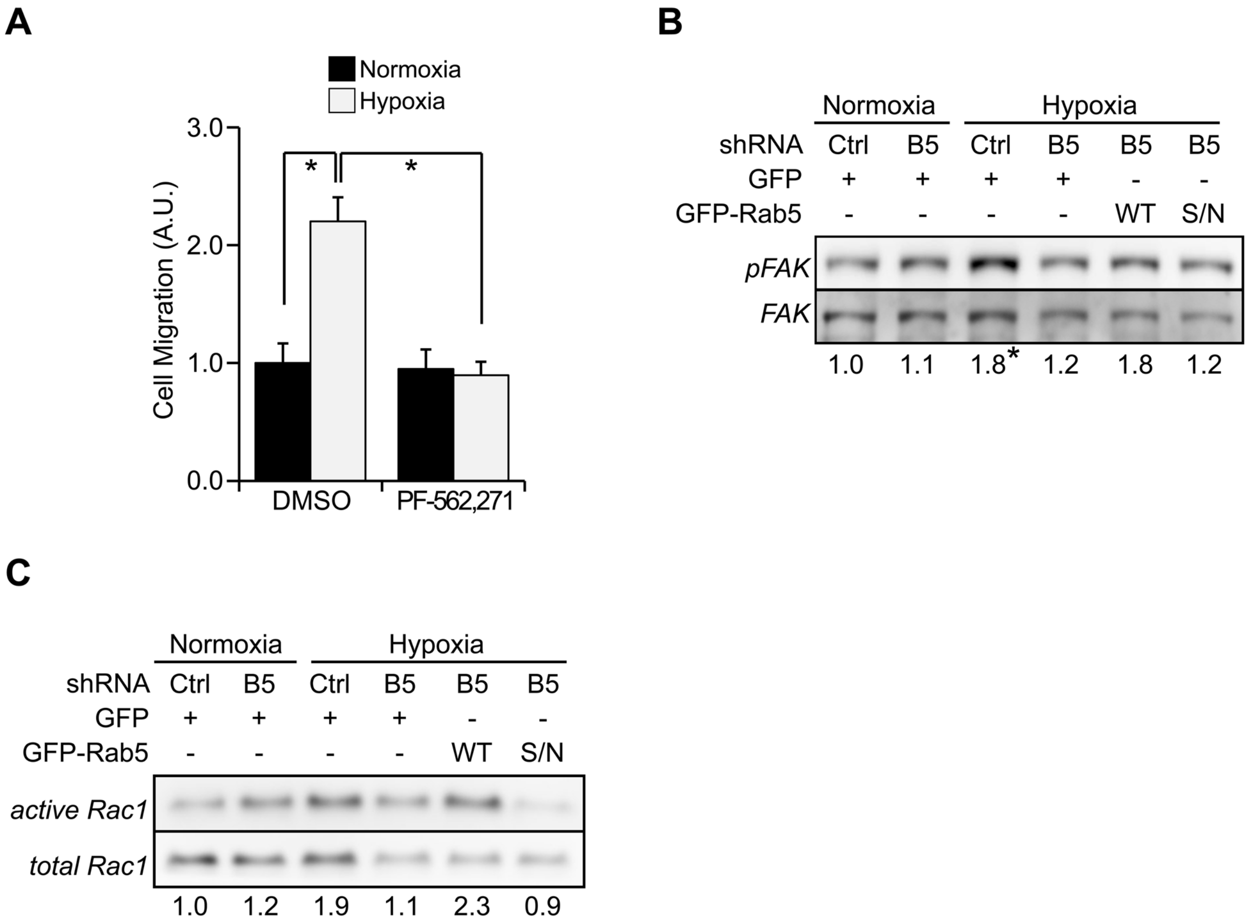

Supplementary Figure S3: A. A549 cells were incubated in normoxia or hypoxia for 24 hours, harvested and then allowed to migrate for 2 hours in Transwell chambers coated with 2 μg/ml fibronectin, in the presence of vehicle (DMSO) or FAK inhibitor (PF-271), under normoxic conditions. Cells that migrated were visualized by crystal violet staining. Data represent the average from three independent experiments (mean ± s.e.m.). *P<0.05. **(B, C)** Cells were transiently transfected with shRNA-resistant constructs, pEGFP-C1 (GFP), pEGFP-Rab5 (WT) or pEGFP-Rab5/S34N (S/N) and incubated in normoxia or hypoxia for 24 hours. **B.** Whole cell lysates were prepared and analyzed by Western blotting with antibodies against FAK and phospho-Y397-FAK (FAK phosphorylated on Y397, pFAK). Representative images are shown. Numerical data below each panel represent the scanning densitometric analysis of phospho-Y397-FAK levels normalized to total FAK from three independent measurements (mean ± s.e.m.) and are summarized as follows: shRNA-Ctrl/normoxia + GFP (1.00 ± 0.05), shRNA-B5/normoxia + GFP (1.04 ± 0.12), shRNA-Ctrl/hypoxia + GFP (1.81 ± 0.10), shRNA-B5/hypoxia + GFP (1.23 ± 0.18), shRNA-B5/hypoxia + WT (1.76 ± 0.34), shRNA-B5/hypoxia + S/N (1.18 ± 0.19). *P<0.05. **C.** Rac1-GTP levels were measured in the GST-PBD pull-down assay. Representative Western blot images are shown. Numerical data below each panel represent the scanning densitometry analysis of Rac1-GTP levels normalized to total Rac1 (mean ± s.d.) and are summarized as follows: shRNA-Ctrl/normoxia + GFP (1.00 ± 0.36), shRNA-B5/normoxia + GFP (1.21 ± 0.11), shRNA-Ctrl/hypoxia + GFP (1.91 ± 0.39), shRNA-B5/hypoxia + GFP (1.13 ± 0.39), shRNA-B5/hypoxia + WT (2.23 ± 1.15), shRNA-B5/hypoxia + S/N (0.85 ± 0.81).

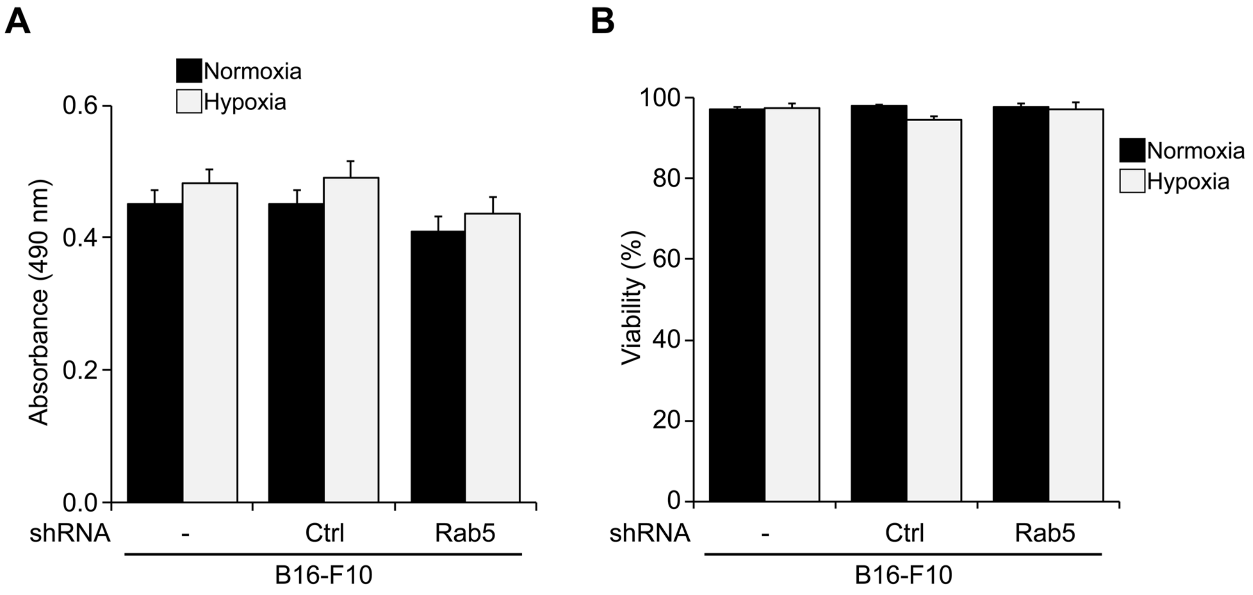

Supplementary Figure S4: A. A549 treated with control or Rab5-targeting shRNA were allowed to grow for 24 hours in normoxia or hypoxia and cell proliferation was measured by the MTS® assay, as indicated in the materials and methods section. Data represent the average from three independent experiments (mean ± s.e.m.). **B.** Cells were grown in the same conditions as indicated in (A) and viability was measured by Trypan Blue Staining, as indicated in the materials and methods section. Data represent the average from three independent experiments (mean ± s.e.m.).

A

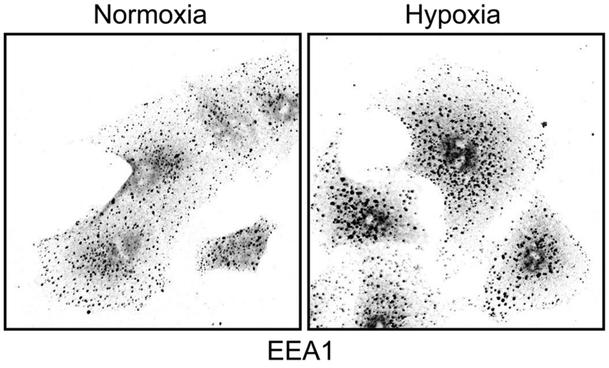

EEA1 Positive vesicles

Area of vesicles (μm²)	Normoxia	Hypoxia
0.05-0.5	76.3 ± 5.3	71.0 ± 4.3 *
0.5-2.0	18.1 ± 4.0	21.9 ± 2.7 *
>2.0	5.6 ± 1.3	7.1 ± 1.6 *

Frecuency (%)

B

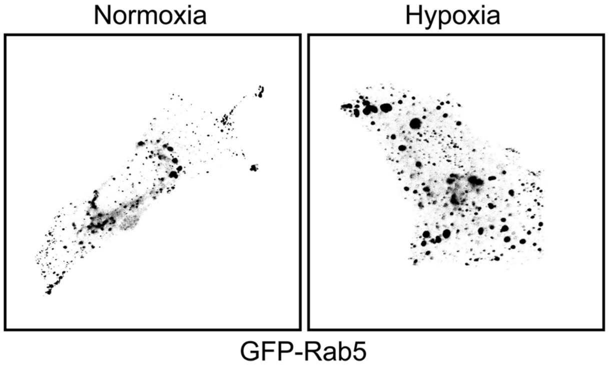

GFP-Rab5 Positive vesicles

Area of vesicles (μm²)	Normoxia	Hypoxia
0.05-0.5	80.9 ± 2.4	78.4 ± 2.8 *
0.5-2.0	15.4 ± 1.2	17.5 ± 2.2 *
>2.0	3.7 ± 1.4	4.0 ± 0.6

Frecuency (%)

Supplementary Figure S5: A. A549 cells were grown on glass coverslips in normoxia or hypoxia for 24 hours. Samples were fixed, incubated with a specific antibody against EEA1 (polyclonal antibody) and analyzed by confocal microscopy. *Left panel*, representative images are shown. *Right panel*, the size of EEA1-positive vesicles was determined with the *Image J* software, as indicated in the materials and methods section. Frequency of vesicle size was grouped in three categories: 0.05-0.5 μm^2, 0.5-2.0 μm^2 and larger than 2 μm^2. Values averaged from two independent experiments (mean ± s.e.m.) are shown. Note that at least 108 cells were analyzed per condition. **B.** A549 cells were grown on glass coverslips, transfected with GFP-Rab5 and then incubated in normoxia or hypoxia for 24 hours. Samples were fixed and analyzed by confocal microscopy. *Left panel*, representative images are shown. *Right panel*, the size of GFP-Rab5-positive vesicles was determined with the *Image J* software. Frequency of vesicle size is presented as indicated in (A). Values averaged from three independent experiments (mean ± s.e.m.) are shown. Note that at least 78 cells were analyzed per condition. *$P<0.05$.
